# Activation of Ca transport in cardiac microsomes enriches functional sets of ER and SR proteins

**DOI:** 10.21203/rs.3.rs-2557992/v1

**Published:** 2023-02-08

**Authors:** Steven E. Cala, Nicholas J. Carruthers, Paul M. Stemmer, Zhenhui Chen, Xuequn Chen

**Affiliations:** Wayne State University; Wayne State University; Wayne State University; Indiana University; Wayne State University

**Keywords:** cardiac sarcoplasmic reticulum, endoplasmic, SERCA, ryanodine receptor, proteome

## Abstract

The importance of sarcoplasmic reticulum (SR) Ca-handling in heart has led to detailed understanding of Ca-release and re-uptake protein complexes, while less is known about other endoplasmic reticulum (ER) functions in the heart. To more fully understand cardiac SR and ER functions, we analyzed cardiac microsomes based on their increased density through the actions of the SR Ca-ATPase (SERCA) and the ryanodine receptor that are highly active in cardiomyocytes. Crude cardiac microsomal vesicles loaded with Ca oxalate produced two higher density subfractions, MedSR and HighSR. Analyses of protein enrichments from the 3 membrane preparations (crude microsomes, MedSR, and HighSR), showed that only a third of microsomal proteins in heart, or 354 proteins, were enriched ≥2.0-fold in SR. Previously studied SR proteins were all enriched, as were proteins associated with canonical ER functions. Contractile, mitochondrial, and sarcolemmal proteins were not enriched. Comparing the levels of SERCA-positive SR proteins in MedSR versus HighSR vesicles produced a range of SR subfraction enrichments signifying differing levels of Ca leak (ryanodine receptor) co-localized in the same membrane patch. All known junctional SR proteins were more enriched in MedSR, while canonical ER proteins were more enriched in HighSR membrane. Proteins from other putative ER/SR subdomains also showed characteristic distributions among SR subpopulations. We conclude that active Ca loading of cardiac microsomes, reflecting the combined activities of Ca uptake by SERCA, and Ca leak by RyR, permits evaluation of multiple functional ER/SR subdomains. Sets of proteins from these subdomains exhibited similar enrichment patterns across membrane subfractions, reflecting the relative levels of SERCA and RyR present within individual patches of cardiac ER and SR.

## Introduction

The basic morphology of the endoplasmic reticulum (ER) in muscle has been known since the initial electron microscopic studies in the 1950’s by Bennett and Porter [1] and Palade and Porter [2], when it was termed the sarcoplasmic reticulum (SR)^1^, given its unique structure that repeats across all sarcomeres in the myocyte. Studies over many decades have focused on its Ca handling properties, beginning with extensive characterization of the important Ca translocating ATPase activity, now termed SERCA, for SR, ER-Ca-ATPase [3, 4]. Much later, a ryanodine-sensitive Ca-releasing activity was identified [5–7]. Extensive analyses of Ca transport in membrane preparations from skeletal muscle also led to established membrane subfractionation protocols, and isolation of membranes of distinct densities that contained different protein composition. Meissner [8] reported the first fractionation of skeletal muscle membranes by isolating a fraction of heavy vesicles that contained the electron-opaque protein polymer calsequestrin [9], and later shown to contain foot processes consistent with morphological depictions of junctional SR [10]. The lighter membranes showed less of the dense luminal protein content, and was concluded to contain free (non-junctional) SR vesicles [8, 9].

A technique for subfractionation of microsomes from heart tissue was later developed that took advantage of the ability of cardiac SR microsomes to concentrate Ca oxalate within their lumens [11–13]. While these studies were originally developed to biochemically separate sarcolemma and SR, subsequent work [5] showed that the Ca oxalate loading had actually produced two distinct types of SR membranes, with roughly half of membrane protein in the densest membrane fraction. And though it was known by then that the drug ryanodine could stimulate Ca accumulation into SR membrane vesicles at high concentrations, these dense membranes were ryanodine insensitive [5].

In contrast, a second membrane fraction of lower density (unable to traverse a 1.5 M sucrose cushion) was highly sensitive to ryanodine [5]. Indeed, by inhibiting Ca leak, 0.3 mM ryanodine added during membrane isolation converted SR membranes of lower density into high density (high Ca oxalate) vesicles [14]. The densities of SR membrane vesicles, as well as the distribution of SR proteins between the two subfractions, are regulated by the relative levels of SERCA and ryanodine receptor (RyR) contained in the fragmented SR patches.

Several studies have used SERCA-positive membrane subpopulations to either validate the identity of a putative junctional SR protein [5, 15–17], or non-junctional SR protein [14, 18–21]. In this study, we determined the complete protein compositions of membrane vesicles generated by SERCA-depending Ca oxalate loading. We found 354 proteins that were enriched as a result of SERCA activation. In addition, a co-enrichment of functionally related proteins resulted from their similar distribution among membrane patches, and reflect the enrichment of ER/SR subdomains in close proximity to SERCA2 and RyR2 activities in intact cardiomyocytes.

## Methods

### Preparation of Crude Cardiac Sarcoplasmic Reticulum Vesicles –

Cardiac microsomes were isolated from female mongrel dog left ventricular tissue, and loaded with Ca oxalate as previously described, with minor modifications [5]. Briefly, heart tissue was homogenized in 10 mM NaCO_3_ at 1:20 dilution (buffer volume/tissue wet weight). Cardiac microsomes were isolated by differential centrifugation, isolating microsomes between 10,000–75,000 × g_max_. In contrast to the previously published method, a final wash of the pellet in 0.6 M KC1, 30 mM histidine, pH 7.0, was not carried out. The investigation conforms to the Guide for the Care and Use of Laboratory Animals published by the US National Institutes of Health (NIH Publication No. 85 – 23, revised 1996). Animal research was approved by the Wayne State University Animal Investigation Committee (protocol #A 04-02-13).

### Ca oxalate loading of SR –

**Figure 1:**
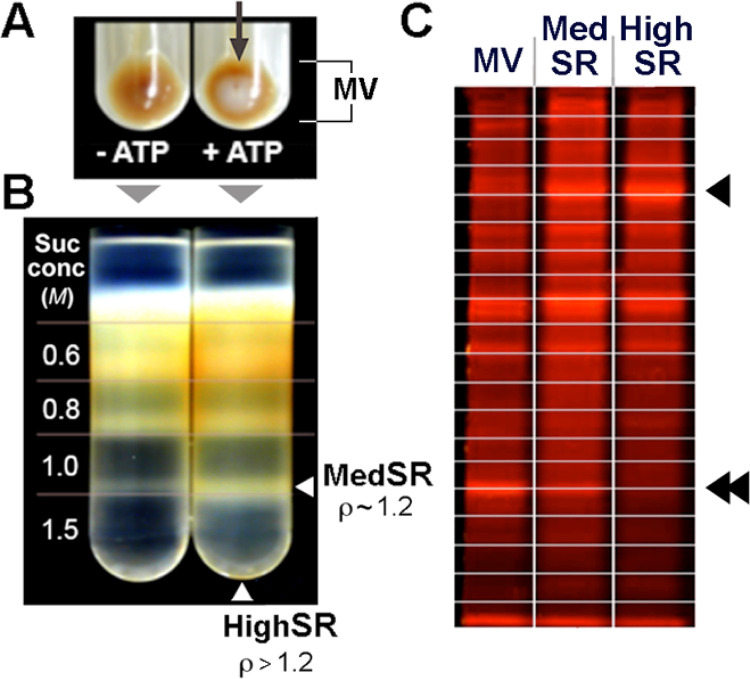
Purification of SR membranes from heart tissue using Ca oxalate loading. ***A***,Crude microsomal vesicles (MV) were isolated by differential sedimentation, then incubated under Ca loading conditions without ATP (−ATP) or +ATP. ***A***, Ca oxalate precipitate inside SR vesicles appears as a whitish pellet, following activation of SERCA2a (*arrow*). ***B***,Separation of MVs on a discontinuous sucrose gradient showed banding of MedSR membrane vesicles afloat on a sucrose concentration (suc conc) of 1.5 M (density r~1.2), and pelleting of more heavily Ca oxalate-loaded HighSR membrane vesicles through 1.5 M sucrose (HighSR pellet not visible in figure). ***C***, 20.0 mg of MV, MedSR, and HighSR protein were fractionated using SDS-PAGE, then trypsinized from 20 separate gel pieces (schematically shown by lines) and analyzed by LC-MS/MS. Even when visualized by protein stain, one sees proteins more enriched in MedSR and HighSR (*arrowhead*) or less enriched (*double arrowheads*).

Loading of crude cardiac microsomes, 75 mg of protein were resuspended in 40 ml of an ice-cold medium containing 50 mM histidine, 100 mM KCI, 65 mM MgCl_2_, 60 mM Na_2_ATP, 25 mM Tris/EGTA, 20 mM CaCl_2_, and 5 mM Tris oxalate (pH 7.1). The suspension was rapidly warmed to 37 °C to initiate active and rapid Ca uptake, and the incubation was conducted for 10 min with 5 mM additional Tris/oxalate added after the first 5 min of incubation. The suspension was then immediately centrifuged at 4 °C for 30 min at 100,000 × g_max_. The resulting brownish membrane pellet included a central white region, indicative of the Ca oxalate precipitate inside the highest density membranes. The whitish center was not present if ATP was not included in the Ca oxalate loading step (Fig. 1A).

### Isolation of medium and high density membrane vesicles after Ca oxalate loading –

Subfractionation of Ca loaded membranes was carried out essentially as previously described [5]. Briefly, membrane pellets were resuspended in 0.25 M sucrose containing 300 mM KC1, 50 mM sodium pyrophosphate, and 100 mM Tris (pH 7.2). This material was layered over a discontinuous sucrose gradient of 0.6 M, 0.8 M, 1.0 M, and 1.5 M sucrose dissolved in the same buffer. After centrifugation at 125,000 × g_max_ for 2 h, membranes were collected from both the 1.5 M sucrose cushion (MedSR) or as the white pellet at the bottom of the gradient (HighSR). These two subfractions were not present in membranes not Ca loaded (Fig. 1B). MedSR was diluted with 4 volumes of ice-cold H_2_O, and then sedimented at 105,000 × g_max_ for 60 min. MedSR and HighSR were resuspended in 0.25 M sucrose, 10 mM histidine, and stored frozen at −20 °C. Protein was determined by the method of Lowry et al. (17).

### Preparation of protein samples for mass spectrometric analysis –

To evaluate the protein composition of the two SERCA-positive membrane fractions, relative to the crude cardiac membranes, we analyzed exactly 20 μg each of MVs, MedSR, and HighSR membranes by SDS-PAGE. The wet gel was stained with SPYRO Ruby red to visualize the protein separations qualitatively (Fig. 1C).

### Tryptic digestion of SR samples –

For in-gel digestion (GeLC-MSMS), each lane (crude cardiac microsomes (MV), MedSR, or HighSR membranes) was cut into 20 gel slices for improved sampling (Fig. 1C). Gel slices were equilibrated in H2O, dried, and digested with trypsin added in dilute solution (1:10 w/w) and digested overnight at 37 °C.

### LC-MS/MS analysis and database search –

Tryptic peptides were separated by reverse phase chromatography (Magic C18 column, Michrom), followed by ionization with the ADVANCE ion source (Michrom), and then analyzed in an LTQ-XL mass spectrometer (Thermo Fisher Scientific). For each MS scan, up to seven MS/MS scans were obtained using collision-induced dissociation. Data analysis was performed using Proteome Discoverer 1.1 (Thermo) which incorporated the Mascot algorithm (Matrix Science). The NCBI database was used against mammalian protein sequences and a reverse decoy protein database was run simultaneously for false discovery rate (FDR) determination. Secondary analysis was performed using Scaffold (Proteome Software). A fixed modification of + 57 on cysteine (carbamidomethylation) and variable modifications of + 16 on methionine (oxidation) and + 42 on protein N-terminus (acetylation) were included in the database search. Minimum protein identification probability was set at ≥ 95%, with 1 unique peptide at ≥ 95% minimum peptide identification probability. From 62,000 individual peptide spectra obtained, we identified 1105 different proteins, along with their relative levels within each of the three different membrane fractions, when levels were sufficient. Raw data and search results have been submitted to the ProteomeXchange via PRIDE with accession PXD022455 (Username: reviewer_pxd022455@ebi.ac.uk, Password: G1wlk66N).

## Results And Discussion

### 1. Protein composition of crude cardiac microsomes

An abundance *index* (A_SR_) was calculated for each protein, calculated as the number of its peptide spectra, divided by protein molecular weight as a rough correction for available target peptides, then normalized to the spectral level for SERCA2, which we defined as A_SR_=100.0. A_SR_ values are a crude surrogate for protein abundance, since factors unrelated to protein abundance will affect the quantifcation of tryptic peptides. Nonetheless, A_SR_ values across sets of functionally related proteins can provide useful comparisons.

In a preparation of cardiac microsomes (MVs), we found 1102 proteins (see Online Resource 1). The most abundant peptides from crude cardiac MV membranes were from known mitochondrial proteins. The three proteins with the highest A_SR_ values in cardiac microsomes were ATP/ADP translocase and ATP synthase, α and β subunits, with known mitochondrial proteins accounted for 7 of the top 10 proteins. The other three major proteins among the top ten highest A_SR_ values were SERCA2, a keratin 1 isoform, and cardiac α1 actin. The focus of our experiments, however, was not directed at crude cardiac microsomal proteins, but instead, at proteins that are enriched in cardiac SR. Of the top 10 microsomal proteins detected, only SERCA2 was enriched in the denser membrane vesicles that result when Ca transport is activated by addition of ATP.

### 2. Enrichment of membrane vesicles by SERCA activation

The two enriched SR subfractions analyzed in this study have previously been described as *junctional SR* and *free SR* vesicles; however, with the much greater number of proteins identified through the use of our GeLC-MS/MS analysis, we have substituted the terms “medium-density SR membranes” (MedSR) and “high-density SR membranes” (HighSR), for these two membrane subpopulations (Table 1). This terminology allows us to discuss the larger collection of proteins, whether or not they participate directly in Ca handling functions.

Comparing the abundance of each protein (A_SR_) in MedSR and HighSR membranes with its abundance in MVs, we determined protein enrichments in SR (E_SR_) (Table 1). Calculated from ratios of A_SR_ values, the enrichment values were relatively unaffected by the sampling issues for any given protein. We used the value of E_SR_ to define *SERCA-positive SR* proteins as those proteins that were enriched by SERCA activation at least 2.0-fold (E_SR_ ≥ 2.0). For 354 SERCA-positive SR proteins, the enrichments varied, but the average enrichment was 8.0-fold (avg E_SR_ = 8.0).

In contrast to enrichment of SR proteins in total SR (HighSR *plus* MedSR), enrichments in HighSR *versus* MedSR membranes (E_sub_), reflect differences in the way small membrane patches are pulled apart, yielding small vesicles with varying levels of Ca release activity (RyR) on top of their very active Ca accumulation. Thus, individual proteins that segregate with *MedSR* were more likely to contain membrane patches that contain RyR; that is, closer to junctional SR sites *in vivo*. To evaluate this distribution as a single number, we defined E_sub_ as the difference between levels in the two SR fractions divided by the total (Table 1). Proteins more enriched in High SR yielded a positive number with a theoretical maximal value of 1.0, and proteins enriched in MedSR yielded a negative number with a theoretical maximal value of −1.0. Values actually ranged from + 0.90 to −0.90.

### 3. Characterization of SR proteins based upon enrichments among membrane subpopulations

**Figure 2:**
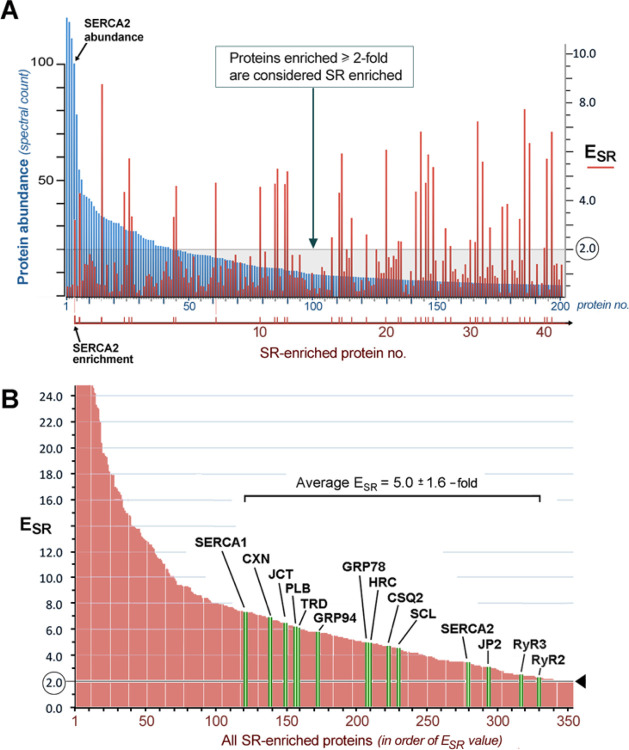
Protein enrichments resulting from Ca oxalate loading of SR vesicles. ***A***, sorting of proteins identified in crude cardiac microsomal vesicles (MV) in order of decreasing peptide abundance, with only the first 200 proteins shown here (*blue bars*). Superimposed is a plot of their fold enrichment in SR fractions (*red bars*). Only about 40 of the 200 were enriched by SERCA activation (E_SR_ = 2.0, *pale gray line*), defining a set of SR proteins, as discussed in this study. The most abundant SR-enriched protein was SERCA2. ***B***, sorting of all 354 SR-enriched proteins by E_SR_ values (which includes the 40 red values in panel A). Shown in green are the E_SR_ values of 14 known cardiac SR proteins, enriched in SERCA-positive membranes on an average of 5.0 ±1.6 (SD) over crude membrane vesicles; 2.0-fold enrichment in SR is marked (*arrowhead*). CXN, *calnexin*; JCT, *junctin plus junctate*; PLB, *phospholamban*; CSQ2, *calsequestrin-2*; TRD, *triadin*; HRC, *histidine-rich Ca-binding protein*; SCL, *sarcalumenin*; JP2, *junctophilin-2*; glucose regulated protein of M_r_=94,000, GRP94.

Of the top 10 microsomal proteins detected in our ventricular muscle sample, only one (SERCA2) was enriched by SERCA activation (E_SR_=3.4). Among 200 microsomal proteins with the highest A_SR_ values (Fig. 2A, *blue bars*), only 40 proteins were enriched at least 2.0-fold by SERCA activation (*red bars*). All SR proteins previously identified as resident to cardiac SR using immunological methods were among the proteins enriched by SERCA activation in our study.

**Figure 3:**
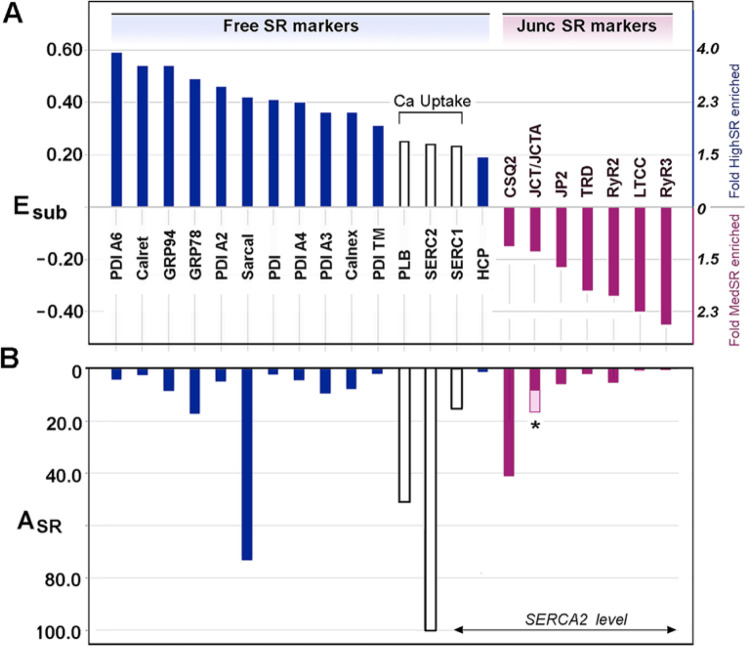
Known cardiac SR proteins distribute between HighSR and MedSR consistent with previous immunological studies. ***A***, well-studied SR proteins exhibit E_sub_ values from +0.60 to −0.45 (*left hand y-axis*), which corresponds to a roughly 4-fold greater level in HighSR membranes to 3-fold greater in MedSR, respectively (*right hand y-axis*). Negative values of E_sub_ based on mass spectrometry data, predict the same SR subdomain distribution described in the literature for 15 non-junctional SR (*blue bars*) and 7 junctional SR (*red bars*) proteins, based on immunological studies. Three SR proteins involved in Ca (oxalate) uptake (*white bars*) were more evenly distributed between SR subfractions. ***B***, Relative abundances for the same 22 proteins show far greater variability. The A_SR_ value for junctin/junctate (JCT/JCTA) is shaded in two halves (*asterisk*, *) to approximate the portion that corresponds only to junctin. PDI, *protein disulfide isomerase*; Calret, *calreticulin*; GRP94, *glucose regulated protein of M*_*r*_=*94,000*; SCL, *sarcalumenin*; Calnex, *calnexin*; HRC, *histidine-rich Ca-binding protein*; PLB, *phospholamban*; CSQ2, *calsequestrin-2*; JP2, *junctophilin-2*; TRD, *triadin*; LTCC, *L-type Ca channel a-subunit*.

For 14 of the best characterized SR proteins (Fig. 2B, *green bars*), the average fold enrichment in SR over crude cardiac microsomes (E_SR_) was 5.0 ±1.6 (mean ±S.D.). When sorted based on their relative distribution between SR subcompartments (E_sub_), all of the known junctional SR proteins were more enriched in MedSR (Fig. 3A), consistent with the idea that MedSR membrane patches probably contain more RyR molecules, possibly combined with less SERCA. Luminal ER/SR proteins, on the other hand, were highly enriched in HighSR (except for calsequestrin-2). SERCA2, SERCA1, and phospholamban were more equally distributed among the membrane vesicles, consistent with a more equal activation of Ca loading in both SR populations. Despite the similar enrichments of known SR proteins, their relative abundances (A_SR_, Fig. 3B) varied greatly, consistent with data widely known from protein staining of SDS-gels [15, 18].

**Figure 4:**
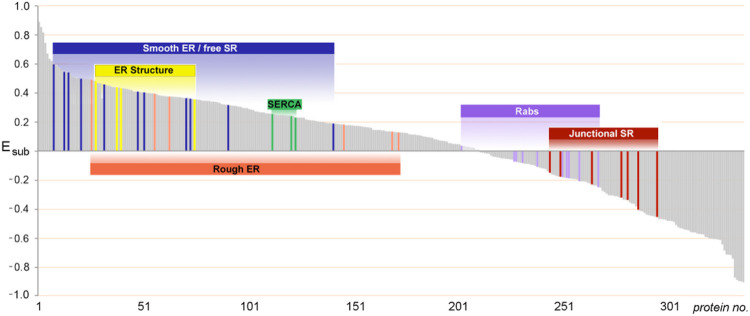
Distributions between HighSR and MedSR are similar for sets of proteins with similar functions. E_sub_ values for all 354 SERCA-positive SR proteins (*shaded gray area*) range from +0.90 to −0.90 (90% more enriched in HighSR or in MedSR, respectively). Distribution of any one protein between the two density layers reflects in part, its physical proximity to junctional SR sites. Co-enrichment of proteins with similar function within common membrane fragments therefore reflect similar relative levels of (nearby) SERCA2 and RyR.

For the entire set of 354 proteins in SERCA-positive membranes, E_sub_ spanned a continuous range of values from + 0.90 to −0.90, corresponding to proteins at a 90% greater level in HighSR, or in MedSR, respectively (Fig. 4). Interestingly, for sets of proteins with related function, E_sub_ values were clustered together. 2, in support of the idea that membrane fragments are generated from ER/SR membrane patches that contain characteristic ratios of SERCA and RyR levels.

### 4. High abundance proteins illustrate wider functions in cardiac SR than only Ca handling

**Figure 5:**
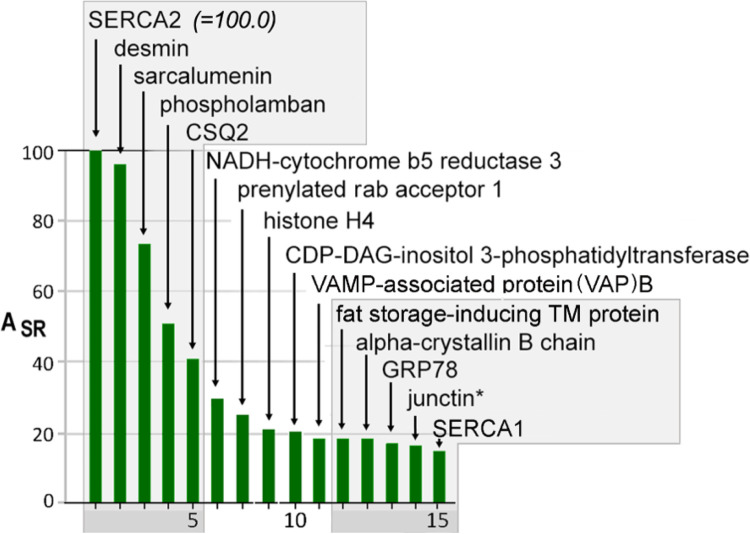
The 15 most abundant SR proteins based on A_SR_ values. A_SR_ values are expressed as a percentage of SERCA2 A_SR_ (=100.0). An asterisk denotes the fact that junctin and junctate were not distinguishable in this study. The 15 proteins shown represented 38% of all spectra from all 354 proteins in SERCA-positive membranes.

A_SR_ values are based upon the number of peptide spectra attributed to a particular protein, but are only semi-quantitative, as they assume similar coverage of every protein sequence by LC-MS/MS, which does not occur [22]. Yet, it was very interesting to look at the proteins with the highest A_SR_ values, as it illustrates the scope of protein functions in cardiac SR. The 5 most abundant proteins in our SERCA-positive SR sample were SERCA2, desmin, sarcalumenin, phospholamban, and calsequestrin-2 (CSQ2), accounting for about a quarter of SR protein mass (percent of total spectra) (Fig. 5). SERCA2a and phospholamban are well known major constituents of cardiac SR [5, 13], and, as expected, their very high A_SR_ values were a predictable consequence of their activity in Ca oxalate loading of SR vesicles. Sarcalumenin is a known luminal constituent of cardiac SR membranes [18, 23], but of uncertain function. It is comprised of two well known splice variants: one generally defined by its roughly 150-kDa apparent molecular weight on standard SDS-gels, and one defined by a roughly 53-kDa [24]. Desmin, a muscle-specific intermediate filament protein of striated muscle [25], was found at A_SR_ levels comparable to SERCA2 (Fig. 5).

Surprisingly high A_SR_ values were also found for many proteins barely discussed in the cardiac research literature. For example, NADH-cytochrome b5 reductase gave the highest A_SR_ value of a non-traditional cardiac SR protein (*A*_*SR*_ = *30.0; 6th highest*). Salviati et al. [26] in 1981 reported very high levels of this redox enzyme, as well as cytochrome b5 itself (*A*_*SR*_ = *5.1; 69th highest*), in the SR of slow skeletal muscle. A prominent escort protein for rab proteins, PRA1 (prenylated rab acceptor 1), was prominently detected (*A*_*SR*_ = *25.0, 7th highest*). The presence of abundant histone H4 (*A*_*SR*_ = *21.0, 8th highest*), and other less abundant histones (Table 2, *Appendix*) may suggest a role as a secreted antimicrobial protein [27, 28] or copper reductase enzyme [29].

The high level of CDP-diacylglycerol-inositol 3-phosphatidyltransferase, isoform 1 (*CDITP, A*_*SR*_ = *20.7, 9th highest*), was consistent with early studies by Kasinathan and Kirchberger [30]. Interestingly, the SR protein with the next highest A_SR_ (VAP-B) is a prominent intra-organellar tethering protein that also plays a putative role in the transfer of synthesized PI to the plasma membrane. Both VAP-B and VAP-A (*A*_*SR*_ = *18.5 and 11,3, 10th and 24th highest, resp*.) were among multiple ER tethering proteins present [31–39] (Table 3). The combined actions of these abundant ER/SR proteins may promote new plasma membrane sites for PI signaling activity in heart, as well as sites for junctophilin-2 binding and junctional SR formation [40–43]. Extended synaptotagmins, detected at low A_SR_ levels, can also transport glycerolipids between the ER and plasma membrane bilayers via a unique lipid-harboring domain [44].

Also yielding a high A_SR_ value was fat storage-inducing transmembrane protein 2 (FIT2) (*A*_*SR*_ = *18.5, 11th highest*), which plays an important role in the formation of lipid droplets and the distribution of lipids between the ER membrane and lipid droplets [45–47]. Alpha crystalline B (*A*_*SR*_ = *18.3, 12th highest*) occurs *in situ* as globular polymers [48]. In total, five prominent filamentous proteins co-enriched with SERCA-positive membrane: desmin and crystalline αB, vimentin 12, keratin II 6B, and beta-tubulin 2C (Table 4, *Appendix*).

### 5. Functional sets of proteins in cardiac ER/SR

The identification of sets of proteins related to known SR and ER functions, added consistency and context to the 354 proteins found in our analysis. Proteins involved in related ER/SR functions were present as complete sets in SERCA-positive membranes. And, while these ER/SR subdomains were present in widely varying abundances (A_SR_ values), their enrichment factors (E_SR_ and E_sub_) were remarkably similar, consistent with their distribution together in membrane patches (vesicles after homogenization). The vast majority of these 354 proteins have not been previously identified in cardiac SR. We briefly summarize the major functional sets of proteins found, and compare their constituent protein enrichments and relative abundances.

#### 5.1 SR Ca pumping

The most abundant peptide spectra, not surprisingly, were from SERCA2. Peptides from phospholamban, a subunit of SERCA2a that dissociates with PKA-dependent phosphorylation were detected at roughly 50% of SERCA2. The need for inclusion of the MW factor to the calculation of A_SR_ values can be easily appreciated in this case, with the 20-fold difference in mass between SERCA and phospholamban molecules. While the relative levels of SERCA2 and phospholamban from this single mongrel canine heart sample will be subject to error and biological variability, the set of three proteins that function in Ca pumping (SERCA2, phospholamban, and SERCA1) were the 1st, 4th, and 15th highest ASR values, accounting for roughly 11% of spectra, supporting the view that Ca accumulation is a primary function of the cardiac ER/SR. Their enrichments in SR (E_SR_), and distribution between MedSR and HighSR membrane subcompartments (E_sub_) were also highly similar (Fig. 5 and Online Resource S1).

#### 5.2 Intraluminal ER/SR proteins

Intraluminal proteins represented an abundant set of proteins based on their number and high A_SR_ values, constituting about 12% of total SR protein, with about half of the mass due to sarcalumenin and calsequestrin (Table 5). Many luminal SR proteins are those carrying a C-terminal sequence Lys-Asp-Glu-Leu (KDEL), which interacts with KDEL receptors to retrieve these proteins from the Golgi back to ER compartments [49–52]. Protein disulfide isomerase (PDI) isoforms, although not previously identified in heart tissue, were among the proteins most enriched in SR (Table 5); also, more highly enriched in HighSR than MedSR (Fig. 3A) consistent with the common notion that free SR is essentially cardiac smooth ER, and that it contains less RyR [53].

#### 5.3 Junctional SR proteins

**Figure 6:**
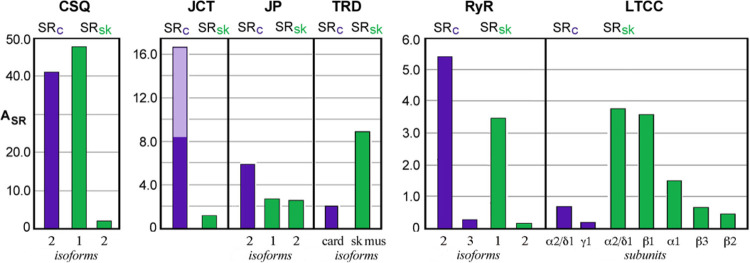
A_SR_ values for well known junctional SR proteins from a preparation of cardiac or skeletal muscle SR. Levels of individual proteins are normalized to that of either SERCA2a (=100.0) for cardiac (Card) SR, or to the levels of SERCA1 (=100.0) for rabbit fast-twitch muscle (Skel) SR proteins as reported in ref. [56]. Note differences in ordinate scales for the three panels shown. Skel data was obtained from a similar proteomic analysis by Liu et al. [56] using the classic preparation of heavy SR prepared from rabbit fast-twitch skeletal muscle that is based upon microsome sedimentation rates [8]. RyR, ryanodine receptor; CSQ, calsequestrin-2; JCT, junctin (plus junctate for Card data); TRD, triadin (cardiac isoform for Card); JP, junctophilin; LTCC, L-type Ca channel. Text below bars designates isoforms or subunits. Double shading of Card JCT denotes the approximate half of peptide spectra that may have arisen from junctate.

The Ca oxalate loading method was previously used to identify several major junctional SR proteins, including calsequestrin-2, cardiac triadin, junctin, and RyR2 [15, 16, 54, 55]. These 4 major junctional SR proteins, along with junctophilin-2, all presented here with relatively high levels of A_SR_, accounting for roughly 5% of total SR peptide spectra. Levels of couplon proteins reported in rabbit fast-twitch skeletal muscle SR [56] show interesting parallels (Fig. 6). Junctin, a splice variant of junctate and aspartyl β-hydroxylase [57], could not be distinguished based on the peptides sequenced. Treves et al. [58] previously reported that no gross differences occurred immunologically between junctin and junctate levels in heart homogenates, suggesting that junctin may be roughly half of the A_SR_ level reported here. Co-enrichment of L-type Ca channel with junctional SR markers was further evidence of a stable protein complex of couplon proteins with the sarcolemmal T-tubule membrane [21].

#### 5.4 Peroxisomal proteins and rab proteins in MedSR

**Figure 7:**
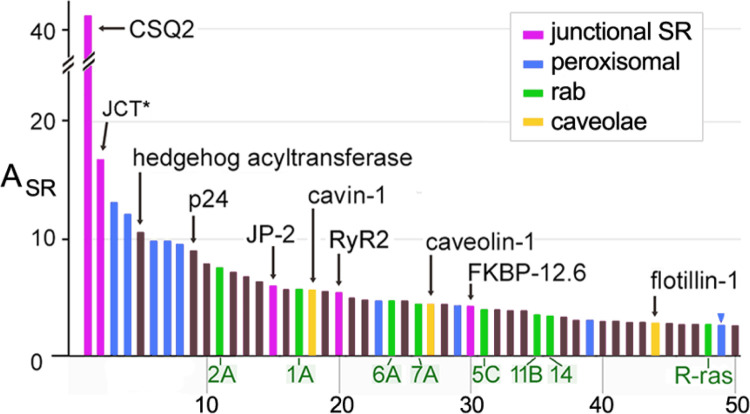
Junctional SR and other ER/SR subdomains enriched in MedSR membranes. SR proteins with negative E_sub_ values (more enriched in MedSR) were sorted by A_SR_ (highest to lowest). Only the 50 highest A_SR_ values are shown. Rab isoforms were an abundant set of enriched proteins (*green bars with labeled isoform*). The total peptide spectra for JCT plus junctate is shown here (JCT, *asterisk* *).

**Figure 8:**
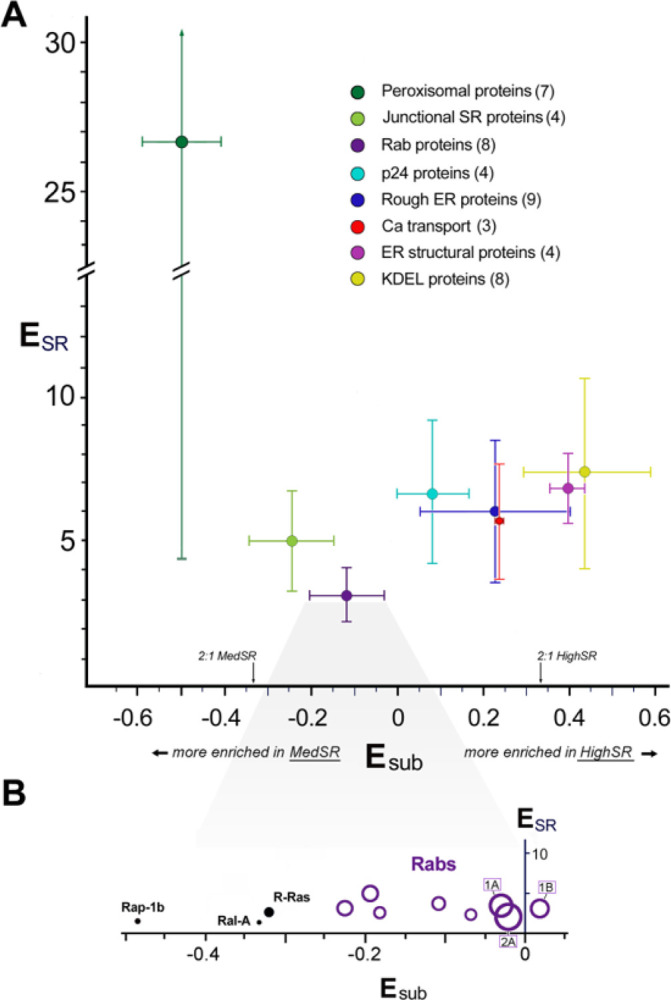
Sets of functionally related proteins undergo similar enrichments ***A***, Subsets of ER/SR proteins with related functions: average enrichment in SR (E_SR_ mean ± S.D., *y-axis*) and average distribution between SR subfractions (E_sub_ mean ± S.D.), *x-axis*. The numbers shown in parentheses are the number of proteins averaged from each set. ***B***, E_SR_ and E_sub_ values for individual rabs (*purple rings*) and other small GTPases are shown. Abundance values (A_SR_) are proportional to symbol diameters. The 3 most abundant rabs are labeled (2A, 1A, and 1B).

In addition to the enrichment of junctional SR proteins, MedSR membranes were also enriched in peroxisomal proteins, rab proteins, and caveolar proteins (Fig. 7), but the three types of protein-containing vesicles varied in their membrane enrichments. Peroxisomal proteins were very uniquely enriched in SERCA-positive membranes, and their enrichment was highly variable (E_SR_=26.7 ±22.3) (Fig. 8A). In addition, peroxisomal proteins were extraordinarily enriched in MedSR compared to HighSR membranes (avg E_sub_ = −0.50 ±0.09). By comparison, junctional SR proteins were much less enriched in MedSR compared to HighSR (avg E_sub_ = −0.24 ±0.10, for 4 couplon proteins). The reason for this unusual enrichment of peroxisomal proteins in SERCA-positive membranes is unknown, and much remains to be learned about this peculiar ER subdomain [60–62].

When E_sub_ values were averaged for several sets of functionally related proteins, average E_sub_ values covered a range of distribution between the two SR subpopulations (Fig. 8A), suggesting that individual membrane patches are enriched in separate functional subdomains, with each subdomain exhibiting particular Ca transport properties that reflect its inclusion of SERCA and RyR protein.

Rabs and other small GTPases were also highly enriched in MedSR, exhibiting E_sub_ values similar to those of known junctional SR proteins (Fig. 8A, B). Rab proteins are fundamental regulators of organelle biogenesis and vesicle transport, and constitute the largest subset of the ras superfamily of small GTPases [63]. In addition to abundant rabs, the rab associated protein PRA-1 (*A*_*SR*_ = *25.0, 7th highest*), and hedgehog acyltransferase-like protein (aka mitsugamin-56 [64]) were also enriched in SR (Online Resource 1). The possible close physical proximity of rabs to junctional SR sites is consistent with evidence of protein secretion in cultured cardiomyocytes emanating from sites close to junctional SR [65].

#### 5.5 Rough ER proteins –

Cardiac rough ER is a critical subdomain of ER, and in cardiomyocytes has a predominantly perinuclear morphology that is distinct from the repeating SR sarcomeres that control contraction [65, 66]. Yet, SERCA-positive SR membrane vesicles also contained a complete collection of known rough ER proteins involved in translation, translocation, and N-linked glycosylation (Table 6, *Appendix*). Rough ER proteins were of relatively low abundance (A_SR_= 4.0 ± 1.7), but very highly-enriched over crude MVs (E_SR_= 6.0 ± 2.5) (Fig. 8A).

#### 5.6 Lipid metabolism and lipid modifications of proteins –

**Figure 9:**
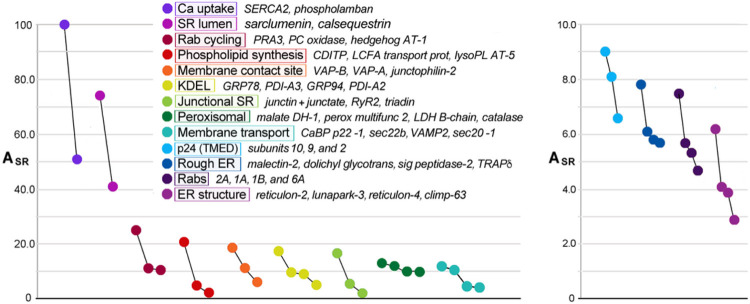
The 2–4 most abundant ER/SR proteins in each functional subset. A_SR_ values sharply decline for the first 2–4 protein of each functional set. Plots are offset along the x-axis to improve visual clarity. A_SR_ values for junctional SR proteins only include transmembrane proteins (cf. Fig. 6), excluding calsequestrin-2 which is grouped with the very abundant sarcalumenin as two very abundant luminal proteins of uncertain function. Proteins with common functions are represented in color-coded sets using similar color tags. Proteins are also color tagged in the table of all proteins (Online Resource 1).

Numerous enzymes involved in lipid metabolism were enriched in SERCA-positive membranes. The highest A_SR_ value (= 20.7) resulted from CDITP, which appends inositol-3-phosphate to diacylglycerol, with numerous lipid metabolizing proteins present at lower A_SR_ levels (Fig. 9, *Appendix*).

#### 5.7 Proteins involved in ER membrane structure and dynamics –

Many cardiac ER/SR proteins are those thought to play roles in distributing and trafficking proteins across the biosynthetic pathway. Several act by guiding membrane patches along transport filaments; these include Ca-binding protein p22, vesicle-trafficking protein Sec22b, vesicle-associated membrane protein 2 (VAMP-2), and vesicle transport protein Sec20 [67–70]. The mammalian proteins of the p24 family (TMED 10, 9, 2, 1) are involved in selective loading of cargo in transport vesicle between membrane compartments, and the co-enrichment and relative abundances of its known subunits support a role in cardiac ER/SR protein distribution [71, 72]. Finally, other ER proteins may function in the maintaining the structure of ER subcompartments, such as reticulons-2 and − 4 [73], lunapark-3 [74], and climp-63 [75]. Proteins involved in ER/SR dynamics are tabulated, along with enrichment values, in Table 7, *Appendix*.

## Conclusions

In this study, we used the technique of Ca oxalate loading of cardiac SR membrane vesicles to produce two SR membrane subpopulations (MedSR and HighSR), thereby enriching patches of cardiac ER/SR membrane that contain combinations of SERCA *and* RyR levels. Roughly one third of microsomal proteins were enriched in SERCA-positive membranes, and about a third of those were more enriched in the MedSR membranes, indicating the presence of enriched RyR in the same SR patch, and suggesting a relative proximity to junctional SR, or at least a biochemically distinct membrane subdomain. The activation of SERCA activity in our current study, an historical measure of SR function, led to the enrichment of proteins from every ER subcompartment, supporting a view that cardiac ER and SR should cannot be demarcated based only upon their ability to function in Ca handling.

Proteins enriched in SERCA-positive SR vesicles were defined as any protein that was enriched ≥ 2.0-fold over its level in crude heart microsomes. This single enrichment criterion selected 354 proteins of 1102 total proteins in crude microsomes, while excluding all mitochondrial, contractile protein, and other known organellar contaminants. Even major mitochondrial and contractile protein contaminants were eliminated by this simple measure of enrichment. SERCA-positive SR proteins encompassed proteins from all known functional ER and SR subdomains, leading us to conclude that SERCA-positive membranes represent cardiac ER and SR. Plotting A_SR_ values for the top 2–4 proteins of different groups of proteins (Fig. 10), provided some semi-quantitative insight into how different ER/SR subdomains may contribute to overall SR function.

In spite of these substantial variations in A_SR_ among functional sets of proteins, the enrichment properties E_sub_ and E_SR_ among the same sets were remarkably consistent; both because of their physical segregation in membrane vesicles, but also because enrichment values are derived from *ratios* of A_SR_ values in two preparations. For example, a large number of known KDEL proteins were found in SR, exhibiting a wide range of A_SR_ values (6.3 ±9.0, N = 7 proteins). For the same 7 KDEL proteins, however, E_sub_ values were 0.47 ±0.08, and E_SR_ values were 7.1 ±3.7.

The enrichment of junctional SR proteins in MedSR membranes was previously demonstrated by immunoblot analyses to be a feature of this SR preparation [5, 15, 18, 21, 76]. In the present study, we found that the values of E_sub_ for 354 proteins formed a continuous range from − 0.9 (largely detected only in MedSR) to + 0.9 (largely detected only in HighSR membranes) (Fig. 4). Other functional protein groups exhibited similarly segregated values (Figs. 3,4,8), suggesting that they too were physically distributed into at least two divergent subcellular sites: those closer to junctional SR and those further removed from such sites. The segregation of protein functional groups in terms of E_SR_ and E_sub_ shows that vesicles are derived from small enough membrane surfaces to fractionate with different enrichment patterns, and do not simply represent huge sections of membrane surface.

In summary, enrichment of cardiac membranes by Ca oxalate loading leads to the enrichment of ER/SR proteins, distributed between membrane fractions that differ in Ca leak through RyR. The distribution of individual proteins between the two fractions (Esub), and the consistent enrichments found among different functional sets of proteins, reflects the connections between ER/SR subdomains and the well-studied spatial relationships between junctional and free SR. Our data present for the first time a reliable estimation of cardiac ER/SR protein content, along with a semi-quantitative assessment of prominent sets of functional ER/SR subdomains present in a microsomal preparation of canine ventricular tissue.

## Figures and Tables

**Table 1:** Key definitions and abbreviations used in this study

Membrane preparations		
MV	Crude cardiac microsomal vesicles	
MedSR	Medium density Ca oxalate loaded SR, enriched in every junctional SR marker	
HighSR	High(est) density Ca oxalate loaded SR, enriched in free SR (non-junctional) vesicles	
Parameter determined for each protein		Range of values
E_SR_	Enrichment in SR (*MedSR + HighSR)/ MV*	≥ 2.0 for SR (average: 8.0-fold)
E_sub_	Enrichment in HighSR compared to MedSR (*HighSR - MedSR)/ (HighSR + MedSR)*	= 0.90 (90% higher in HighSR) = −0.90 (90% higher in MedSR) = 0.00 (equally distributed)
A_SR_	Spectral Abundance in SR *(MedSR + HighSR)*, divided by molecular weight, normalized to SERCA2 *(= 100.0)*	0.05–100.0 *A*_*SR*_ *(SERCA2)* ≡ *100.0*

**Table 3:** Membrane contact site (MCS) proteins enriched in cardiac ER/SR MCS proteins are sorted by ASR values, and their rank order among 354 proteins that are in SERCA-positive SR (Rank). High values of A_SR_ for VAP-B and VAP-A reflect the importance of cardiac ER/SR in transporting proteins and lipids to distal parts of the myocyte. Other proteins involved in ER inter-organellar contact include junctophilin-2, and extended synaptotagmins. Only junctophilin-2 showed a negative E_sub_ value, consistent with its reported enrichment in junctional SR (cf. Figure 3A).

Membrane contact site protein	Rank	A_SR_	E_SR_	E_sub_	Role
VAP-B (vesicle-associated membrane protein (VAMP)-associated protein B)	10	18.5	5.6	0.40	Associated with VAMP subfamily of SNARES; involved in lipid transfer from ER on lipid transfer proteins.
VAP-A	24	11.3	7.4	0.29	
junctophilin-2	55	5.9	3.1	−0.23	Binds to PI enriched plasma membrane lipids (MORN sites) at junctional SR.
extended synaptotagman-2	288	0.55	5.7	0.33	Tethers ER to the plasma membrane by C-terminal MCS, and transports glycerolipids between the two bilayers.
extended synaptotagman-1	308	0.37	3.1	0.13	

**Table 5:** Proteins of the ER/SR lumen Resident luminal ER/SR proteins, listed in order of decreasing ASR. Proteins known to be highly specific for cardiac and skeletal muscle myocytes cells are indicated (*shaded rows*) [18]. All other proteins likely act as ER protein chaperones, and are maintained in ER through a C-terminal –KDEL retrieval signal (asterisk*). Calnexin is a Type I transmembrane protein with a large luminal segment that has calreticulin-like chaperone activity [59]. GRP78, glucoseregulated protein, M_r_ = 78 kDa; PDI, protein disulfide isomerase; His-rich Ca BP, histidine-rich Ca binding protein. Rank identifies proteins by their order among 354 proteins in SERCA-positive SR sorted by A_SR_ (see Online Resource 1).

Protein	KDEL	Rank	A_SR_	E_SR_	E_sub_
sarcalumenin		3	73.2	4.6	0.42
calsequestrin		5	41.1	4.7	−0.15
GRP78	*	13	17.3	5.0	0.49
PDI A3	*	32	9.6	6.2	0.36
GRP94	*	36	8.5	5.7	0.54
calnexin		41	7.7	6.8	0.36
PDI A2	*	73	4.9	5.1	0.46
PDI A4	*	91	4.3	7.8	0.40
PDI A6	*	94	4.2	15.2	0.59
calreticulin	*	142	2.4	8.5	0.54
PDI	*	145	2.3	7.0	0.41
PDI isoform TMX	*	159	2.1	6.8	0.11
His-rich Ca BP		217	1.2	5.0	0.19

## Data Availability

The datasets generated during and/or analyzed during the current study are available at Figshare.com; Filename: CardiacSRproteome; DOI: 10.6084/m9.figshare.19953701. Raw data and search results have been submitted to the ProteomeXchange via PRIDE with accession PXD022455 (Username: reviewer_pxd022455@ebi.ac.uk, Password: G1wlk66N).
